# Effects of racism on the socio-emotional wellbeing of Aboriginal Australian children

**DOI:** 10.1186/s12939-019-1036-9

**Published:** 2019-08-22

**Authors:** D. M. Macedo, L. G. Smithers, R. M. Roberts, Y. Paradies, L. M. Jamieson

**Affiliations:** 1 ARCPOH, AHMS Building Level 9, The University of Adelaide, North Terrace, Adelaide/SA, 5005 Australia; 2 BetterStart Child Health and Development Research Group, School of Public Health, The University of Adelaide, Adelaide, Australia; 3 School of Psychology, The University of Adelaide, Adelaide, Australia; 4 Alfred Deakin Institute for Citizenship and Globalisation, Deakin University, Melbourne, Australia

**Keywords:** Racism, Social and emotional wellbeing, Mental health, Aboriginal Australian children, Childhood

## Abstract

**Background:**

Racism is a pervasive experience in the life of Aboriginal Australians that begins in childhood. As a psychosocial stressor, racism compromises wellbeing and impacts developmental trajectories. The purpose of the present study was to estimate the effect of racism on indicators of Australian Aboriginal child socio-emotional wellbeing (SEWB) at one to two years after exposure. Age-related differences in the onset of symptoms were explored.

**Methods:**

Data from the B- and K-cohorts of the Longitudinal Study of Indigenous Children were used (aged 6 to 12 years). Racism, confounding variables, and the Strengths and Difficulties Questionnaire (a measure of SEWB) were collected by questionnaires and guided interviews with each child’s main caregiver. Adjusted Poisson regression was used to estimate the relative risk (RR_a_) effects of racism on SEWB for both cohorts separately. RR_a_ were pooled in a random effects meta-analysis.

**Results:**

Exposure to racism was associated with an adjusted point estimate indicating a 41% increased risk for total emotional and behavioural difficulties, although the confidence intervals were wide (pooled RR_a_ 1.41, 95% CI 0.75, 2.07). Analyses by cohort showed younger children had higher RR_a_ for total difficulties (RR_a_ 1.72, 95% CI 1.16, 2.54), whilst older children had higher RR_a_ for hyperactive behaviour (RR_a_ 1.66, 95% CI 1.01, 2.73).

**Conclusions:**

The effects observed contributes to our understanding of the impact of racism on Aboriginal Australian children. Support for emotional and behavioural difficulties, and hyperactive behaviour, for Aboriginal children might help counteract the effects of racism. Future longitudinal research and policies aimed at reducing racism in Australian society are necessary.

## Background

The concept of racism corresponds to a set of attitudes, behaviours and practices that maintain an imbalance in the distribution of power across ethnic-racial groups [1]. Racism is the oppression of specific ethnic-racial groups in association with maintaining the privileges of others, fostering and perpetuating social disparities [2]. At an institutional level, racism can be observed through historical and structural inequalities in socioeconomic indicators, and educational and health parameters. In its interpersonal facet, racism permeates daily interactions, with negative discriminatory behaviour targeted at ethnic-racial minority members. Racism can be internalised by the assimilation of negative messages that influence self-concept formation and well-being [1–3]. Interpersonal racism is the focus of the present study, as such experiences are reported by members of different ethnic-racial minority groups at different ages across the life-span, with documented impacts on health and wellbeing [4, 5]. Accordingly, racism will be the term used throughout this paper in reference to racially-based discriminatory encounters experienced at an interpersonal level. The impact racism has on ethnic-racial minorities’ health and wellbeing makes it a public health issue and a central component of the political agenda worldwide [4].

The World Health Organisation framework to strengthen health equities globally and within countries is based on the social determinants of health [6]. This framework highlights how social stratification influences early life and the social and physical environments in which individuals develop and interact [6, 7]. Among these structural factors, biases and values within society, social position, ethnicity and race, and psychosocial factors are central determinants of the distribution of health and wellbeing in the population [6]. This framework has been suggested as relevant to the understanding of health and wellbeing inequalities in the Aboriginal Australian context [8]. Aboriginal people report being exposed to racism during childhood, adolescence, and adulthood, in a range of different settings where they perform their daily activities [9–11]. Accordingly, Aboriginal health and wellbeing cannot be promoted without considering the impact of the social and structural conditions that shape their life trajectories [8]. Due to its link to a history of disposession, marginalisation, disempowerment, and inequalities, racism is a central determinant of the health and socio-emotional wellbeing of Aboriginal Australians from an early age [8, 12].

The effects of racism among ethnic-racial minorities can be observed from childhood and early adolescence [11]. A systematic review of 121 studies offers a panoramic perspective of the main findings in the field. It indicates that the most consistent effects of racism among children and adolescents are for negative mental health outcomes [13]. Associations between racism and lower self-esteem and higher anxiety, stress, depression, suicide ideation and attempts, aggression, Attention Deficit and Hyperactive Disorder, and behavioural problems have been reported [13]. Similar associations were reported in a systematic review that analysed 47 papers on the health and wellbeing of Indigenous youth (4–20 years) from high income countries (U.S., Canada, and Australia) [14]. More specifically, research with Aboriginal Australian adolescents and young adults indicates associations of racism and poor overall mental health and higher anxiety, depression, and suicide risk [15, 16]. A study focused on 5–10-year olds showed an increased risk for overall emotional and behavioural difficulties amongst Aboriginal children exposed to interpersonal racism [17].

Research in the Aboriginal Australian context is still limited, generally reporting cross-sectional data and including relatively large age-range groups [15].The present study intends to provide further insight on the impact of racism on the social and emotional wellbeing (SEWB) of Aboriginal Australian children. Following on from the previous work of Shepherd et al. [17], we used longitudinal data to estimate the effect of racism on specific domains of SEWB including emotional difficulties, hyperactivity, peer and conduct problems, as well as a total score for psychological difficulties. We hypothesised that experiencing racism would be associated with higher risk for clinically significant symptomatology in all domains considered one-to-two years after exposure. Estimating the effects of racism on different mental health domains can contribute to the understanding of symptoms that might be more closely associated with experiences of racism in Aboriginal children, assisting in identifying periods for optimal intervention and in designing appropriate support.

The analyses were designed to maximize the longitudinal potential of the data. Temporal ordering of exposure and outcome is required to account for the possibility of reverse causation, thus contributing to the investigation of causal associations and the long-term effects of exposure to racism amongst racial minorities [13, 17]. Age-related differences in the onset of symptoms were also investigated. Differences in the onset of symptoms might be relevant for mental health clinicians, health practitioners, and professionals in educational settings who interact with Aboriginal children and might have opportunities to identify symptomatology and recommend early intervention.

## Methods

### Data collection procedures

Data were from the Footprints in Time - Longitudinal Study of Indigenous Children (LSIC). Initiated and funded by the Australian Government Department of Social Services (DSS), the LSIC is a national survey focused on gathering data on determinants of Aboriginal child physical and mental health. Its main goal is to provide information on how experience in the early years can affect Aboriginal children health and development [18]. The LSIC employs a cross-sequential design involving two cohorts of children. The Baby Cohort (B Cohort) comprises Indigenous children born between December 2006 and November 2007, with data collection commencing when children were aged 6 months to 2 years. The Child Cohort (K Cohort) includes children aged 3.5 to 5 years in the first wave. They were born between December 2003 and November 2004. We used data from both cohorts of LSIC to maximise our sample size. The study commenced in 2008 with subsequent waves conducted annually thereafter [18].

The first stage of sampling was the selection of 11 sites across Australia representative of the socioeconomic and community environments where Aboriginal children lived. Locations ranged from very remote communities to capital cities. A non-representative purposive sample was recruited using addresses provided by Centrelink and Medicare Australia of children registered as Aboriginal or Torres Strait Islander. Centrelink is part of the Australian Government Department of Human Services, which delivers social security payments for those unemployed or unable to work [19]. Medicare Australia provides benefits, payments, and services to assist all Australians with the costs of health services, prescriptive medicines, and medical equipment [20]. Children were also recruited via recommendation of study families and local knowledge of Research Administration Officers involved in the study. Promotion of the study occurred during community events such as National Aboriginal and Islander Day Observance Committee week [21].

Eligible families were thus approached, and voluntary consent obtained. The probability of being selected to participate was not random across the total Australian Indigenous population, neither were children and families selected at random within each specific location [18]. Data were collected through questionnaire-guided interviews conducted by trained Aboriginal and Torres Strait Islander Research Administration Officers. Information was collected from multiple informants (e.g., study child main caregiver, main caregiver’s partner, study child, study child teacher) [22].

### Participants

Data on 1060 Aboriginal children participating in waves 6, 7, and 8 of LSIC were used. Data collection occurred in 2012, 2013, and 2015 respectively. The children included in the analysis were aged 6 to 12 years. The number of interviews conducted ranged from 1239 to 1255 between the three waves and the study participants’ retention rate ranged from 85 to 87%. The information used was provided by the self-identified main caregiver of the child, usually the mother (86% in the K Cohort and 87% in the B Cohort).

### Variables measurement and categorization

#### Study child experience of racism (exposure)

Information on racism experienced by the children in school was obtained through the question “Has study child been bullied or treated unfairly at school because he/she is Aboriginal and/or Torres Strait Islander?” Answer options were “Yes, bullied (kids being mean to him/her”, “Yes, treated unfairly (adults being mean to him/her)”, “Yes, both bullied and treated unfairly”, “No”, “Don’t know”, and “Refused”. In both cohorts, only one answer pointed to discriminatory treatment by adults. The item “Yes, both bullied and treated unfairly” was endorsed by 7 and 12 participants in the K and B cohorts, respectively. As it was not possible to discriminate if the perpetrators reported in this item were peers or adults, answers were dichotomized in “Yes” and “No” as to reflect overall experiences of racism. The “Don’t know” and “Refused” responses were classified as missing data. To verify the effect of racism over time, the exposure was collected at wave 6 (K cohort) and wave 7 (B cohort). Children were aged 6.5 to 8 years (B Cohort) and 8.5 to 10 years (K Cohort) when information on racism was obtained. No information on the timing of racism exposure was specified by the question.

#### Children socio-emotional wellbeing (outcome)

The Strengths and Difficulties Questionnaire (SDQ) [23] scores were used to assess risk for clinically significant emotional or behavioural difficulties, or difficulties that might require further investigation for mental health-related diagnosis. Comprising 25 questions, the SDQ assesses difficulties in five domains: emotional symptoms, conduct problems, hyperactivity, peer problems and pro-social behaviour. Scores range from 0 to 10, with higher scores indicating higher difficulties; reverse applied to the pro-social behaviour domain. The scores of the emotional symptoms (anxiety, emotional withdrawal, somatic complaints), conduct problems (rule breaking, emotional outburst, defiant behaviour), hyperactivity (inattention, impulsivity, restless), and peer problems (relationship with other children, experience of being bullied) were used separately to analyse domain-specific difficulties. A SDQ Total score was the sum of all domains (excluding Prosocial), with scores ranging from 0 to 40 (higher scores indicating higher socio-emotional problems).

SDQ scores were collected at wave 8, dated from 2015, when children were 7.5 to 12 years old. All scores were dichotomised based on cut-off points for high risk of emotional or behavioural difficulties, based on a UK sample of 10,438 5–15 year-old children [24]. Scores ≥5 were considered at risk for emotional symptoms, ≥4 for both conduct and peer problems, ≥7 for hyperactivity, and ≥ 17 for the total SDQ score. These cut-off points represents the children above the 90th percentile in the Meltzer et al. [23] sample, suggesting elevated risk of presenting emotional and behavioural difficulties. The SDQ is a widely used measure to assess child risk for emotional and behavioural difficulties across a different range of countries and contexts [24]. The acceptability, face, and construct validity of the SDQ has been demonstrated among urban Aboriginal children and adolescents in New South Wales, Australia [25, 26]. No specific cut-off scores for Aboriginal Australian children are available.

#### Confounding variables

Demographic characteristics and socio-economic status were used to adjust for potential bias due to confounding. Confounders were conceptualised as a common cause of the exposure and the outcomes [27]. Confounders were selected based on content-knowledge and literature-based evidence amongst racial minority children, including Aboriginal Australian children [13, 28, 29]. Child sex, child age (years), main language spoken by the study child, main caregiver level of education, the Index of Relative Indigenous Socioeconomic Outcomes (IRISEO), and level of relative geographic isolation were included in the adjusted models. Sex and age were provided in wave 1. The dominant language spoken by the Study child was derived from the question “What language (s) is Study Child learning?” Responses included English, foreign languages, and a set of Aboriginal languages (e.g. Alyawarr, Pitjantjatjara, Yorta-Yorta). The final categories of the variable were “Equally fluent in English and in an Indigenous language”, “Dominant in an Indigenous language” and “Dominant in English”. Information on children’s dominant language was collected at Wave 8 and is considered to approximate the language proficiency in previous waves.

The main caregiver highest level of education attainment was collected at waves 2 and 3 with fourteen response options ranging from “Never attended school” to “Post graduate degree”. Caregiver education data was collected from waves 6 and 7. Participants responses were recategorized in four categories: “Year ten or below of high school”, “Year 11 or 12 of High School”, “Post school certificate/Advanced diploma”, “Graduate degree or above”. Socioeconomic status was adjusted for using the Index of Relative Indigenous Socioeconomic Outcomes (IRISEO), a measure of community level socioeconomic advantage. Based on the 2006 Australian Census of Population and Housing, the measure is derived from information on education, housing, and income and is calculated specifically for Indigenous Australians. The measure presents continuous values ranging from 1 (most disadvantaged) to 10 (most advantaged) [30]. The Level of Relative Isolation (LORI) is a measure of geographic remoteness/isolation based on the Accessibility/Remoteness index of Australia, which in turn is calculated based on relative distance to service centres. The LORI categories range from “No isolation”, which corresponds to metropolitan areas to “Low isolation”, “Moderation isolation”, “High isolation” and “Extreme isolation” [31].

#### Analytical approach

Descriptive analytical procedures were used to obtain estimates of the frequency distributions with confidence intervals (CI) for each cohort separately. Risk Ratios (RR_a_) and 95% CI were calculated from Poisson regression analysis with robust errors to estimate the effects of child exposure to racism on socio-emotional wellbeing for each cohort. Models were adjusted for confounding, as above. Multiple imputation with chained equations (MICE) were conducted for each cohort separately to address potential bias due to missing information. It also accounted for the loss of precision and statistical power resulting from the exclusion of participants with incomplete information [32]. MICE was conducted under the assumption that missing values occurred at random, conditional on the observed data [33]. Models of imputation included all the exposure, outcomes, and confounding variables used in the association models. The variables with complete data were the same in both cohorts: child age; child sex; IRISEO; and LORI. The variables with missing values in the K Cohort were: SDQ scores (1); parental education (7); racism (9); and child dominant language (18). The same variables presented missing values in the B Cohort: SDQ scores (4); racism (9); parental education (33); and child dominant language (40). Twenty data sets with imputed values were generated to reduce sampling variability from the imputation process [34]. After imputation, the association model tested from the K cohort included 412 observations. The model tested from the B Cohort included 648. The descriptive estimates of prevalence (95% CI) generated represent the frequency distribution of data across the twenty imputed data sets. No final N per variable was informed as they vary between data sets, as is the case of analyses using imputed data. The results from the imputed analysis were considered the primary findings for each cohort.

Children in the K Cohort were two years older when information on racism was collected (wave 6) and had SEWB assessed after a two-year interval (wave 8). The children in the B Cohort were assessed for racism at wave 7 and their SEWB after a one-year interval (wave 8). The pooled effect estimates were analysed as an average measure of the effect of racism on child SEWB. A single parameter nonetheless is limited in summarising heterogeneous effects [35]. Thus, the meta-analysis allowed examination of consistency across the effects in two different-aged cohorts that had different times of assessment for exposure and outcomes and thus had different opportunities to be exposed and develop symptoms. This was considered more appropriate than combining both samples.

The focus of our analysis was effect sizes as an indication of the impact of racism on child SEWB and their precision. As recommended by the American Statistical Association and the American Psychological Association, we do not interpret statistical significance [36, 37]. Thus, no *p* values are reported, and the CIs are interpreted as measures of precision and not containing a true effect in the population [37, 38]. We understand the limitations of our study and report all information for these results to be included in any future meta-analysis on estimating the effects of racism, assisting the research in the area to move forward [37]. All analysis were performed in Stata 14.

## Results

The mean ages of children at measurement of racism in the K and B cohorts were 8.5 (SD 0.57) and 6.6 years (SD 0.54), respectively. Mean age when the outcome was assessed was 10.5 (SD 0.58) and 7.6 years (SD 0.56), respectively. The data in Table 1 describes the two cohorts and illustrates that the cohorts were similar for most variables. There were slightly more female children, corresponding to 52% of participants in both waves. English was the child’s dominant language in 90% of cases for both cohorts and the IRISEO means were 5.8 (SD 2.21) and 5.7 (SD 2.54) for the K and B cohorts respectively. The highest level of caregiver education attainment was high school or below for approximately 60% of the two samples and at least three quarters of each of the two cohorts presented low to no levels of relative geographic isolation. Exposure to racism was similar across both cohorts (K Cohort, 15%; B Cohort, 14%), as was the proportion of children with clinically-significant symptoms on the SDQ Table 1.

**Table 1:** Descriptive distributions of exposure, outcomes and confounding variables for waves 6 and 7

Means (Standard Deviations)	K Cohort	B Cohort
Child Age	8.5 (SD 0.57)	6.6 (SD 0.54)
IRISEO	5.8 (SD 2.21)	5.7 (SD 2.54)
Prevalence (95% CI)	K Cohort	B Cohort
Racism		
Yes	15.3 (11.8, 19.0)	14.0 (11.2, 16.6)
No	84.7 (81.2, 88.2)	86.0 (83.3, 88.7)
Socioemotional wellbeing		
SDQ – High Emotional symptoms scores	14.8 (11.3, 18.2)	18.3 (15.3, 21.3)
SDQ – High Conduct problems scores	17.9 (14.2, 21.6)	22.4 (19.2, 25.6)
SDQ - High Hyperactivity scores	17.7 (14.0, 21.4)	20.7 (17.6, 23.9)
SDQ – High Peer problems scores	20.1 (16.2, 24.0)	20.1 (17.0, 23.2)
SDQ – High Total difficulties scores	14.5 (11.1, 17.9)	18.1 (15.1, 21.1)
Sex		
Male	47.4 (44.0, 50.7)	47.2 (43.3, 51.0)
Female	52.5 (49.2, 55.9)	52.7 (48.9, 56.6)
Child dominant language		
Equally fluent – English/Indigenous language	5.4 (2.4, 5.0)	3.8 (2.2, 5.3)
Indigenous language	4.7 (2.6, 6.9)	6.3 (4.4, 8.2)
English	89.8 (86.8, 92.7)	89.8 (87.4, 92.2)
Main caregiver level of education		
Year ten of High School or below	31.5 (27.0, 36.0)	33.8 (30.0, 37.5)
Year 11 or 12 of High School	26.2 (22.0, 30.5)	30.0 (26.4, 33.6)
Post School certificate or Advanced diploma	32.3 (27.7, 36.8)	26.8 (23.2, 30.3)
Graduate degree or above	10.0 (7.0, 13.0)	9.3 (7.0, 11.6)
Level of Relative Isolation		
None	28.3 (24.0, 32.7)	27.4 (24.0, 30.9)
Low	54.3 (49.5, 59.1)	49.7 (45.8, 53.5)
Moderate	9.7 (6.8, 12.5)	15.6 (12.7, 18.3)
High/Extreme	7.5 (4.9, 10.0)	7.2 (5.2, 9.2)

**Fig. 1:**
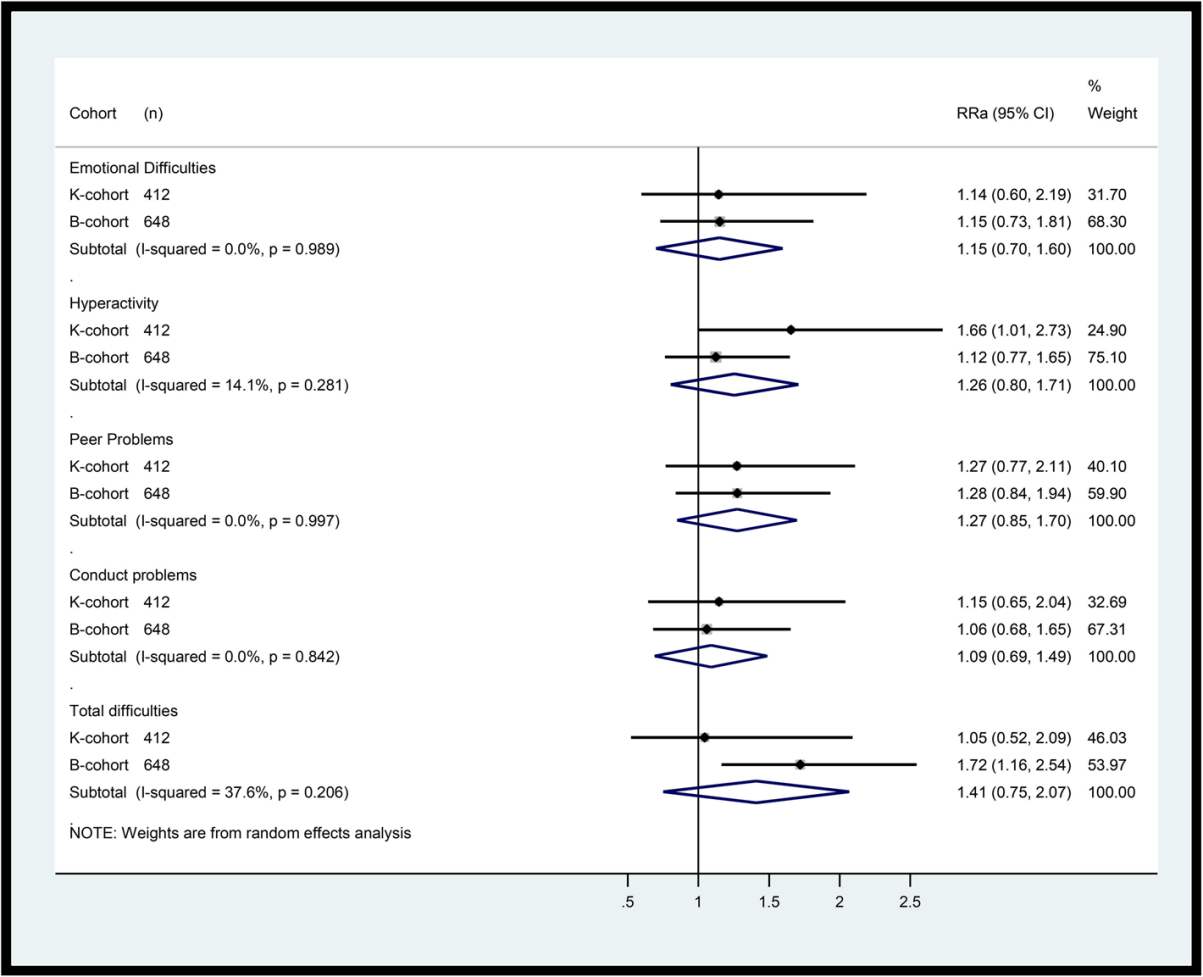
Confounder-adjusted relative risks of racism on socio-emotional wellbeing of Aboriginal Australian children

Figure 1 shows the RR_a_ for each cohort and each domain. The point estimates were consistent for most domains although CIs were generally wider for the K cohort compared with the B cohort. However, in the hyperactivity domain, there was a considerably higher RR_a_ for the K cohort (RR_a_ 1.66, 95% CI 1.01, 2.73) than the B cohort (RR_a_ 1.12, 95% CI 0.77, 1.65). The B cohort in turn presented higher RR_a_ for total clinically significant difficulties (RR_a_ 1.72, 95% CI 1.16, 2.54) than the K cohort (RR_a_ 1.05, 95% CI 0.52, 2.09). The pooled estimates between cohorts showed larger RR_a_ for total difficulties (RR_a_ 1.41, 95% CI 0.75, 2.07), peer problems (RR_a_ 1.27, 95% CI 0.85, 1.70), and hyperactive behaviour (RR_a_ 1.26, 95% CI 0.80, 1.71). The I^2^ for the effects on emotional symptoms, conduct problems, and peer problems was equal to 0%, suggesting the variability of the estimates between the cohorts was entirely due to chance. The observed I^2^ in the hyperactivity and the total difficulties domains suggest that 14.1 and 37.6% respectively of the variability among the estimates of each domain was due to heterogeneity between the cohorts [Insert Fig. 1 near here; Title: ‘Cohort-specific and pooled estimates of the effects of racism on social and emotional wellbeing domains’].

## Discussion

The findings support our hypothesis that experiencing racism in childhood is associated with higher risks of clinically significant symptomatology in all SDQ domains. The risk ratios discussed are the effects with highest compatibility with the models tested. Nonetheless, careful interpretation is required as the null values were also compatible, although less likely. The effects observed might reflect an initial understanding of a psychosocial stressor such as racism and the implied oppression based on ethnic-racial membership. Children in both waves are still in the process of assigning meaning to the racially determined distribution of power in society, as they mature biologically, cognitively, and socially [39]. There is evidence that from early childhood individuals possess cognitive representations of social dominance, expecting that socially dominant individuals will be more competent and have more resources [40, 41]. It is possible that, although some children might be less prone to identifying racism prior to adolescence, there might be an increased risk for the mental health and well-being of those who do [17]. Our results suggest that these effects might persist over time, with the temporal association applied in the analysis indicating that racism may impact children one to two years after initial exposure.

Previous findings using LSIC data demonstrated an associated odds ratio of 2.32 (95% CI 1.52, 3.53) in the total SDQ emotional and behavioural difficulties score for children assessed for racism experiences at ages 5 to 10 [17]. Our results align with these findings. Although the direction of the effect is the same, comparisons regarding its size are limited by the different effect measures presented (risk ratios and odds ratios). We calculated risk ratios rather than odds ratios because the odds ratios are overestimated when the outcome has a close or higher prevalence than 20%, as it is in this case [42]. In that sense, our effect measures are more conservative. Shepherd et al. [17] combined exposure to racism data from four waves of LSIC (between waves 1–6), which ‘averaged’ the effect and potentially obscured age-related effects that we observed in the study reported here. The study follows a research approach focused on the cumulative effects of primary and vicarious racism. No analysis on specific domains of child SEWB –or age-related differences - were reported, not permitting further comparisons [17].

As children age, affective and cognitive maturation might influence how racism is perceived and how its impact manifests [39]. Our results provide an insight into its effects on specific SEWB domains in different age-groups. The children in the older cohort were at increased risk of presenting hyperactive behaviour. A less precise effect was also observed for increased risk of conduct problems. This might indicate that professionals aiming to reduce externalizing symptoms, especially hyperactive behaviour, among Aboriginal children in later years of childhood need to consider the role of racism in the onset of symptoms. Prevalence data from Australian schools suggests that bullying and discrimination tends to increase during middle primary school up until the transition to secondary school [43], which includes the age-range of children in our sample. Strategies to identify and respond to racist episodes might help to reduce such effects and need to be the focus of future research and intervention [44].

The younger children in our study were shown to be at higher risk for total emotional and behavioural difficulties. This suggests the effects of racism were not especially pronounced in a given domain but were observed through different emotional and behavioural difficulties for that age group. Both cohorts showed an increased risk, again less precise, for the onset of emotional difficulties. Such effects demonstrate how children in both age-groups might present anxiety, emotional withdrawal, somatic complaints and other internalising symptoms due to racism. Comorbidity between the two symptoms’ typology is reported in the literature and explains the effects observed in different domains, as children who are presenting behavioural difficulties are likely to also be experiencing internalising problems [45].

Both cohorts exhibited increased risk for the onset of peer problems, although the poor internal consistency of the SDQ Peer problems scale among Aboriginal Australians [26] indicates its results should be interpreted with care. It is argued that its items might not reflect the importance culturally given to different interpersonal relationships (e..g, relationship with elders and the broader community; importance of kinship) for the wellbeing of a child. Thus, in the Aboriginal Australian context, problems with peers might not be conceptualized as a threat to child SEWB, provided the child has positive relationships with family and community members [25, 26]. Removing the peer relationship subscale, however, did not improve the fit of the original SDQ model, showing it is still appropriate for Aboriginal Australian children [26]. It should be noted that poor internal consistency of the peer problems scale was also observed in other populations [46, 47]. Future qualitative studies are needed to inform the direction of further modifications for using the Peer Problem scale among Aboriginal children [26].

It is important to observe that our sample was assessed for the risk of presenting future clinically significant symptomatology, requiring care for comparisons with clinical diagnoses. Nonetheless, the effects presented here are relevant to primary care practitioners, mental health care providers, and school professionals who work with Aboriginal children. They suggest that Aboriginal children might show emotional and behavioural difficulties as the outcome of experiencing racism. Older children might be especially prone to presenting hyperactive behaviour (e.g., lack of attention, agitated behaviour) and conduct problems (e.g., defiant behaviour, and small infractions).

Aboriginal Australian conceptions of resilience in children include the centrality of culture, connection to country, kinship, and community [48, 49]. Accordingly, promotion of a strong ethnic-racial identity has been shown to be an important component in promoting social and emotional wellbeing among Indigenous youth of Australia, U.S., and Canada [50–52]. Future research and interventions that take into consideration the Aboriginal Australian concepts of wellbeing and resilience might assist in fostering connection to culture and sense of pride about one’s ethnic-racial identity [48]. Research on the effectiveness of ethnic-racial identity in reducing the effects of racism from an early age can inform future policy and intervention [53].

Our results were obtained from a large sample of children of a stigmatised racial minority group in Australia. The children participating in the LSIC are diverse culturally and geographically, with more than 80 Aboriginal or Torres Strait Islander tribal groups (e.g., Wiradjuri, Yorta Yorta, Arrernt, Gamilaroi) being represented [54]. It can thus be argued that LSIC data is one of the best information sources on determinants of the health and development of Aboriginal Australian children, considering the unprecedented number of participating children, the annual follow up, and the sampling covering a range of localities where Aboriginal children live [18, 55].

Compared with the few longitudinal studies on this topic [5, 17], the longitudinal design of LSIC ensured temporal order of the exposure before the outcome. As for other strengths of the study, the SDQ is a valid and reliable instrument for using with different cultural groups, also being the most common tool used in studies involving Aboriginal children [25]. The analysis of effects per domain of SEWB also contributed to understanding which aspects of development might be most sensitive to racism amongst different age groups. For the method’s rigor in estimating the effects of interest, adjustment for confounding was adopted for bias reduction and MICE was performed to reduce non-response bias.

We also highlight that our models were not adjusted for SDQ scores at baseline. First, information at baseline was only available for the K-cohort. Second, our research question was not related to the effects of racism on changes in SDQ scores between waves. Considering the complex dynamics of racism, we cannot be sure that a child’s exposure started at baseline as to justify adjusting for SDQ score at this point in time. We believe that our measure of racism is an approximation of children’s experiences and might reflect an ongoing process. Finally, adjustment for baseline outcomes might reduce certain bias but can introduce others. In a paper published in the American Journal of Epidemiology, Glymor and collaborators [56] argue that the bias introduced can surpass the bias eliminated. It not only fails to remove confounding but also can induces spurious correlations between exposure and measured change. When adjustment for baseline functions is measured prior to exposure, as would be the case for one of our cohorts, such adjustment could introduce regression-to-the-mean bias if baseline values are measured with error [56].

Despite adjusting for several cofounders, residual and unmeasured confounding may remain. Another limitation is the two-year difference between assessment for exposure and outcomes for children in wave 6 (K-cohort), while it was one year for children in wave 7 (B-cohort). Children in the K-cohort, due to their already higher age and the larger time interval between assessments, had more opportunity to be exposed to new episodes of racism that were not captured. Consequently, there may be more children in the K-cohort who experienced a negative impact on SEWB due to racism but who were counted as unexposed, underestimating the effect sizes presented. It could also be the case that the children were continuously exposed to racism in the intervals between assessments, reflecting a cumulative effect when SDQ scores were captured. It is important to note that the exposure variable was racism in the school environment and not in other settings and, as such, this potentially underestimates children’s exposure to racism. It is possible that only the more severe episodes of racism will be reported by children to their parents, which again, would contribute to underestimating racism exposure. Although caregivers believe their children would tell them about bullying/victimization at school, children who suffer discrimination refer not telling their caregivers about such experiences [57]. Therefore, future research should seek to understand experiences of racism from the child’s perspective and across all contexts [13].

All the point estimates of the risk ratios indicated that racism was associated with increased risks of poorer SEWB. However, the CIs were wide and ‘non-significant’. We deliberately avoid interpreting ‘statistical significance’ and focus on effect sizes, as recommended by the leading professional organisations in statistics and the health sciences [37, 38, 58, 59]. Larger sample sizes might address the wide CIs [59]. However, this is the largest cohort available in Australia, and one of few in the world [60, 61], with data available to study effects of racism in childhood. Therefore, it is unlikely that larger samples are available. We felt it was inappropriate to combine data from the B and K cohorts due to differences in the ages when racism and SEWB were measured, and differences in the intervening period (the two cohorts had different opportunities to be exposed). Furthermore, the separation of the two cohorts has added a unique insight that age might influence which aspects of SEWB are affected by racism, which would have been masked if the cohorts were combined. Irrespective, our data from two cohorts are presented in such a way that they could be used in future meta-analyses that aim to more precisely estimate the effect of racism on SEWB.

## Conclusions

The present study demonstrated the effect of racism on the socio-emotional wellbeing of Aboriginal Australian children aged 6 to 12 years. Differences of this effect within subgroups based on age were observed, with important implications for identification of exposure to racism and management of specific symptomatology in children. Neglecting such signs could contribute to the perpetuation of the intergenerational effect of racism experiences. Future research with longitudinal data should be conducted to help elucidate how this symptomatology evolves over adolescence and into adulthood. Although mental health support may be necessary for children’s wellbeing, reduction of racism must be a target of public policies that aim to build a more equal and diverse society for all Australians [62].

## Data Availability

There are security and confidentiality protocols for accessing LSIC data. Interested parts must submit an application and sign a deed of license. Information can be found on the LSIC webpage: http://www.dss.gov.au/lsic.

## Abbreviations

DHS: Department of human services
DSS: Department of social services
IRISEO: Index of relative indigenous socioeconomic outcomes
LORI: Level of relative isolation
LSIC: Longitudinal study of indigenous children
MICE: Multiple imputation with chained equations
SDQ: Strengths and difficulties questionnaire
SEWB: Social and emotional wellbeing
